# Involving Moral and Ethical Principles in Safety Management Systems

**DOI:** 10.3390/ijerph18168511

**Published:** 2021-08-12

**Authors:** Paul Lindhout, Genserik Reniers

**Affiliations:** 1 TPM Safety & Security Science Group (S3G), Delft University of Technology, 2628 BX Delft, The Netherlands; linteu@paal39.nl; 2 KULeuven, Campus Brussels-Center for Corporate Sustainability (CEDON), 1000 Brussels, Belgium; 3 Faculty of Applied Economic Sciences and Engineering Mgmt (ENM), University of Antwerp, 2000 Antwerp, Belgium

**Keywords:** safety management system, ethical principles, misconduct, transgression, precautionary, fairness, prevention

## Abstract

Some organisations, and some individual humans, violate moral and ethical rules, whether or not they are written down in laws or codes of conduct. Corporate transgressions, as this behaviour is called, occur because of the actions of those in charge, usually bright and dedicated people. Immoral and unethical conduct can adversely affect the safety of workers, the general public and the environment. A scoping review method for a literature search is used to explore morality and ethics in relation to health and safety management. Our findings show that controlling the risks associated with misconduct and corporate transgression is not usually seen as a responsibility allocated to safety systems but is left to general management and corporate governance. The moral and ethical principles, however, can be applied in safety management systems to prevent misconduct and transgression-related safety risks. Our results show that ethical leadership, ethical behaviour, sustaining an ethical climate and implementation of an ethical decision-making process emerge as key preventive measures. The discussion presents a proposed way to include these measures in safety management systems. Conclusion and recommendations underline that unwanted behaviour and transgression risks can be brought under control, starting from a set of best practices. Not only the managers themselves but also board members, independent external supervisors and government regulators need to embrace these practices.

## 1. Introduction

In some companies and institutions, the people in charge break generally accepted rules. This conduct can have adverse effects for health, safety and the environment. In practice, the associated risks are not controlled within company safety management systems and are therefore often regarded, by default, as a direct responsibility of the general management. Against the background of morality and ethics, we explore the nature and possible consequences for health and safety of violations of broadly accepted rules. Based on literature review we identify the best practices and then propose to include countermeasures in safety management systems.

When someone is brusque, impolite or condescending towards someone else, this violates unwritten social rules. The recipients of such behaviour will most likely choose to avoid any further dealings with this individual. The other option would be to file a complaint and find an authority with the ability to enforce proper conduct. 

Abuse, deceit and scam are more complicated to deal with for an individual. Avoiding such intentionally performed damage is difficult for the disadvantaged person. The costs and effort of filing a lawsuit may not even lead to compensation of the damage. Theft and extortion are regarded as a crime and the criminal justice system and insurance companies need to be involved. However, obtaining compensation for the damages suffered can be unattainable for many reasons. Both perpetrator and victim are individuals in such cases. The perpetrator engages with immoral conduct, pursuing personal interests, to the disadvantage of the victim.

Comparing this conduct with transgressing businesses yields an amazing resemblance. There, companies and large corporations are the perpetrators and the general public, the employees, the environment, the government and regulatory institutions are the victims. Transgressing companies may for example take unacceptable risks, damage the environment, cause harm to people, charge high prices to customers, deliver poor quality products and provide substandard, insecure and unsafe workplaces to their personnel. Such self-serving and immoral company behaviour is similar to that of a person. These violations of moral and ethical rules, *corporate transgressions*, often are the result of the actions of bright people acting on what they believe to be to their company’s best interest or their own interest [1,2,3].

## 2. Problem Definition

So how can people fall off the right track? People use their moral compass to navigate through life. Research has shown that this compass originates from culture. This leads to moral values and then to a personal set of social and ethical norms, creating someone’s individual moral and ethical standard. People normally make their decisions respecting the standard. In exceptional cases however, some people might decide to disengage from a part of the set of norms, e.g., with the prospect of great material gain or an increase in power, they allow themselves to engage in immoral conduct.

If people become involved in corporate transgressions, not only the quality of their own work can be affected but also that of others. They too can be disengaged from moral and ethical standards. Research shows that this can, either intentionally or accidentally, compromise the company safety performance [4,5].

Current safety management systems, often built around a core structure described by Hale [6] and intended to control risks according to the principles laid down in the ISO-31000 standard [7], do not include moral and ethical aspects. The ungrateful role of a whistle blower often is all that remains for those who notice the misconduct or transgression but are not part of it themselves.

Safety managers need an established and accepted way to take remedial action when they notice a transgression and suspect the presence of an uncontrolled safety risk. This study centres on the following question:

*In what way should moral and ethical principles be included in safety management systems?*

## 3. Method

The scoping review method [8] is chosen to create an overview of current boundaries for safety management in companies of generally accepted moral and ethical principles in society, and of types of corporate transgressions. The latter are screened for health and safety management-related content. In support of this a literature search is used and a selection process according to Byrne [9], Daykin and Coad [10]. Because of the general nature of the research question, Google Scholar and its associated proprietary databases were used. A series of consecutive searches were performed with different combinations of the search terms:

“ethic(s)(al), moral, safety, science, occupational, environmental, health, value, disengagement, norm, principle, risk, management, system” and with: “fairness, equity, precautionary, cautionary, industry”s.

Since the subject at hand is an aspect of a slow-pace development process, different time periods were used to include the development of safety management systems since the nineteen seventies. Reference listings were screened for further sources. Next, the sources identified on title and abstract were deselected or admitted on the basis of relevance to the research question and on their scientific quality. Several non-academic secondary sources and grey sources were admitted because of their particular relevance [11,12].

The admitted sources were screened on content related to several subjects: morality and ethics in current safety management practice, moral and ethical principles in society, principles relevant to health and safety, ethical concerns in safety management, kinds of misconduct and transgressions, and a set of existing countermeasures from practice.

A simple text analysis and meta-synthesis method was then used to structure the data gathered per subject. This method can be used to compare and aggregate findings from multiple sources in order to establish an overview of common viewpoints, new interpretation and new knowledge [13].

Based on the set of measures, a way to include control of the misconduct and transgressions-related risks in safety management systems is proposed.

## 4. Results

### 4.1. Literature Search Process Results

**Figure 1:**
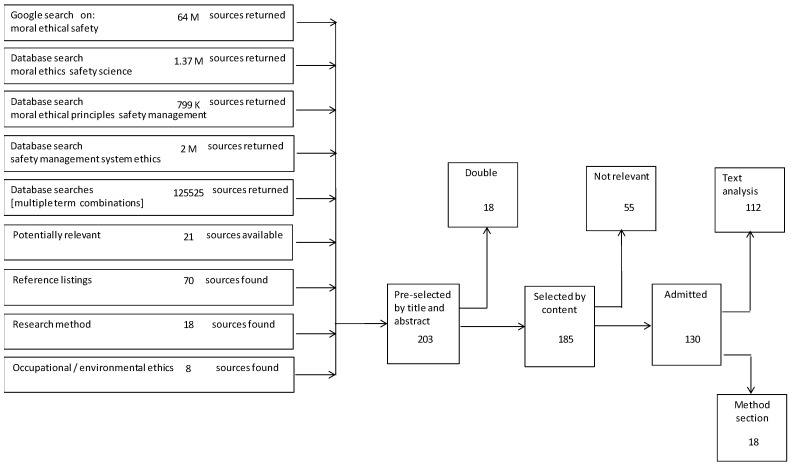
Literature search and selection process results showing the numbers of sources in each step.

Nine searches were conducted, as indicated in Figure 1, each leading to a number of sources returned. Using the selection process described in the method section these led to admission of 130 sources. Of these, 112 sources were subjected to text analysis and meta-synthesis [13], of which the results are presented in the following sections.

Figure 1 shows the literature search and selection process results.

### 4.2. Ethics in Current Safety Management Practice

Safety managers normally work within the boundaries set by the management philosophy implemented in a company. In practice, their job description often includes the requirement to abide corporate policy, to comply with local, national and international legislation and to respect the governmental permit to work conditions applicable to the company premises. Written or unwritten codes of conduct may be applicable. A safety professional is usually recruited on the basis of technical knowledge, previous experience in practice and a set of social skills [14]. Professional standards [7,15,16], a desired personal style [17,18], company safety targets and safety culture objectives then complete the safety manager’s operational framework [14]. In the well-established area of occupational health and safety, however, very few studies address the practical implementation of ethics [19,20]. In the increasingly important field of environmental health ethics, ethicists have different views and use conflicting approaches when it comes to the environment and nature [21].

Moral and ethical principles, including those concerning health and safety, are normally considered to be a part of business ethics [22], or of corporate ethics management [23] or of corporate social responsibility [24]. In contrast to this, *non-controversial values* have a presence in safety management practice, yet tend to be overlooked [25]. This situation excludes moral and ethical principles, at least partially, if not completely, from safety management responsibility. Moral or ethical misconduct by individuals and corporate transgressions have the potential to cause uncontrolled, hence unacceptable, safety risk. Hence, this exclusion is relevant to safety management [4,5] since such risks require the implementation of adequate risk control, similar to all other risks [7]. A relation between moral and ethical principles and safety management appears to be both missing and necessary.

### 4.3. Moral and Ethical Principles in a Range of Situations in Society

Both individuals and organizations ought to comply with widely accepted moral and ethical principles in society. In order for any organization to be viable, sustainable, long term profitable and socially responsible, the shareholders, leaders, employees, including safety managers, ought to embrace these principles [22,26]. Findings from general society, governance, government, regulators, organizations, health research, companies, human resources management, software development and health and safety management suggest that the distinction between what is good and what is bad, starts from values [27,28,29]. Values relevant to society are, e.g., the respect for human life, privacy, dignity, freedom, fairness, truth, reciprocity, safety, security and common good [30,31,32,33].

Morality is about the question “what is right?” and about respect for human values, e.g., in bio-medical research: respect for autonomy, non-maleficence, beneficence and justice [19,34,35,36]. Rahanu et al. also mentions fidelity, reparation (undo damage done to others), justice, beneficence, self-improvement, gratitude and non-injury [29]. Resnik mentions protection of animal welfare, stewardship of natural resources, and ecological sustainability [37].

The rights, distributive justice, care and virtue principles are frequently mentioned as moral principles constituting a basis for ethics [38].

In turn, ethics are based on morality [39]. A range of ethical principles is found for the many situations and fields in society, e.g., the inviolability of national sovereignty, social equity, human rights, freedom [22], mutual respect, equality and inclusion [34,40], personal integrity [41,42,43], justice, fairness, non-maleficence, responsibility, privacy, transparency [44,45,46], value for human life, integrity, justice, the common good and excellence [27], justice as fairness [44,47,48], and a principle labelled as the golden rule: reciprocity, i.e., treat others as you like to be treated [29,32]. Most of these ethical principles also affect safety management.

Ethical principles lead to social norms [49]. Norms themselves influence choices people make, also in their behaviour relevant to safety [50]. Laws, rules and codes of conduct prescribe good behaviour, prohibit misconduct and prevent behaviour based on vices rather than virtues [29,39,51]. People can also voluntarily choose for good behaviour on basis of virtue ethics, e.g., altruism, generosity, tolerance, honesty, wisdom [39,52]. Behaviour based on vices, e.g., violence, greed, slander, lust, exploitation, intolerance or selfishness, is considered to be unethical [39]. An organization might voluntarily set itself a societal goal or assume a social responsibility or duty, e.g., human dignity, sustainability, solidarity, magnanimity (use own strength to help others) or quality of life [29,51,53].

### 4.4. Ethical Principles Important for Health and Safety

The need for safety is not being debated now and is not expected to be in the future. As Hollnagel puts it: “*That safety is important needs little argumentation*” [4]. There is a broad consensus on what safety is about. At the core of safety-related ethics is the notion of “*not wanting to see anybody hurt*” [54]. This notion is taken up in several international declarations, charters and covenants. For an individual, no harm, health and safety are declared human rights [46,55,56,57,58]. From a set of eight government principles for risk control, three touch upon ethics, addressing criteria for risk acceptance, transparency in risk communication and equity [59]. For health and safety in particular, cautionary, precautionary, equity and fairness are mentioned as ethical principles [25].

In industry, several initiatives were taken to develop and implement a set of principles for ethical conduct. The UN Global Compact initiative [60] mentions ten principles: protection of human rights, non-complicity in abuse, free association, no forced labour, no child labour, no discrimination, precautionary approach to the environment, responsibility for the environment, environmentally friendly technology and no corruption.

An emerging set of eight ethical corporate conduct standard principles was identified by [22]. At the Caux Round Table meeting [61] between international business representatives, respect for stakeholders, societal development, environment and performing beyond the requirements set by law, were included in their seven agreed business principles. All this leads to an indicative comparison between societal and institutional ethical principles and emerging corporate conduct principles, see Table 1. Many of these principles are important for health and safety. The main observation is that the ethical principles embraced by society and adopted by the UN international institutions are currently only partly covered by the set of emerging corporate conduct principles.

The indicative comparison in Table 1, inspired by an earlier study on ethics in occupational health and safety [19], shows that a significant part of the ethical principles is not being addressed in the emerging set of corporate conduct principles. Important for society in general is that the common good, equity and virtue principles are missing. Although a citizenship principle is present in the set, this void suggests that companies focus more on their own interests than on their contribution to society.

Specifically relevant for safety management is that the cautionary, autonomy, and excellence principles are either not addressed or not fully adopted. This means that risk informed consent, continuous improvement activities and ambition to go beyond mere compliance with the law, are not wholeheartedly supported, although a few points are now addressed in the seven agreed Caux standards [61]. This comparison demonstrates that an important part of the four ethical principles relevant to safety management [25] is missing. A detailed review of the principles in the corporate conduct set, which, at first glance, seem to address principles embraced by society and international institutions, results in a range of differences. This shows that the content of principles described in the set falls short with respect to embraced societal and proposed institutional principles.

### 4.5. Ethical Concerns and Issues in Safety Management

Several ethical issues specifically related to health and safety risk management are mentioned in literature: risk-informed employees, cost-benefit analysis (CBA), minimum safety criterion, the precautionary principle and the ALARP principle [62].

There are many more ethical concerns and issues affecting safety though. Frequently mentioned are: the provision of safe and risk resilient workplaces and avoiding societal harm [46], ethical cost-benefit analysis [63,64,65], consent to risk [66,67] and ethical aspects of paying workers extra for more risky work [46,68].

Safety professionals are facing tensions and burdens due to uncertainties, due to lack of knowledge, general management not embracing ethical values, and safety being regarded as a value while facing financial and economic constraints [63,64,69,70,71]. Several questions remain to be resolved on a case-by-case basis, e.g., what criteria are to be used to determine whether a risk is acceptable [63,72] and how far a safety professional should go to protect workers [64].

In practice, safety management faces a complex set of day-to-day problems with moral and ethical aspects. Even full regulatory compliance does not make everything safe [63,65,73] since legislation and standards simply do not cover every activity in industry and society, and since legislation, seen as solidified experience, is lagging behind. Emerging technologies, e.g., nanotechnology, biotechnology, artificial intelligence, autonomous weapons, genomics, big data analytics, and their associated new ethical concerns and risks add new ethical issues [30]. Although risk informed consent obtained from workers is important, it does not relieve employers from their responsibility for workplace safety [67]. Many ethical issues are originating from diversity in the workforce, e.g., in international business and on a multilingual shop floor, or from respecting a local culture, operating under an undemocratic government, in an industry with low wages, or in a social setting with poverty or discrimination [22,64,74]. Of a more general nature is the concern that there is a significant lack of knowledge about ethics and risk in organizations [45,75,76].

The weak relation between safety culture and moral intent [77] and the fact that *complying to moral pressure and ethical norms* does not necessarily make a workplace safe [78], necessitates other improvement actions within safety management systems.

Moreover, administrative practices, such as monitoring compliance, elaborate cost-benefit determinations, risk analyses, and metrics, e.g., on human error or human behaviour, can also constrain or reduce safety [65].

Safety management might be outside of its area of competence when it comes to illegal activities, e.g., corruption, bribery or child labour, but safety management should address responsibility for poor workplace safety, lack of environmental protection, risk taking behaviour [22] and the board of directors’ negligence or recklessness [79,80].

### 4.6. Misconduct and Transgression Types

Several types of misconduct and transgression are reported in literature. Immoral and unethical conduct might be found in many places in organizations relevant to health, safety and environment, e.g., providing unsafe workplaces [46], using poor research study designs or fabricated data [66], euphemistic labelling [81], presenting false information about services [82], paying workers more to let them follow a more risky and more profitable procedure [68], underestimating or ignoring risks [83], creating incentives for unethical conduct [52], and pursuing personal gain [84]. Five groups of wrong behaviour of individuals and organizations emerge from the literature findings in this study: abuse of power, misleading information, reduced moral awareness, risk of exposure and fraud.

#### 4.6.1. Abuse of Power

Individuals can engage in self-interest that “*often takes the form of pursuing personal, material, or status-related gains*” [84]. Besides gain for the self, there are exploitation or oppression of others and “dehumanization in outcomes” [46], e.g., abuse of their job insecurity [85].

#### 4.6.2. Wrong or Misleading Information

Both individuals and organizations can engage in working with faulty study design, wrong test subjects, too few samples, poor statistics, fabricated results, misinterpretation, biased conclusions [66], administrative process flaws [86], poor risk assessments, wrong handling of uncertainty, misinforming stakeholders and poor uniformity [83]. Examples are: false descriptions of goods or services, false price indications, less weight than stated being delivered, poor food safety, substandard product quality, poor hygiene and incorrect labelling [82].

#### 4.6.3. Reducing Moral Awareness

Organizations can engage in reframing of moral consequences of damaging behaviours and dampening moral awareness among employees, which is used to reduce the uneasy feeling among workers when a company is transgressing [87]. This effect can be achieved by using Bandura’s eight mechanisms of moral disengagement: 1, moral justification; 2, euphemistic labelling; 3, advantageous comparison; 4, displacement of responsibility; 5, diffusion of responsibility; 6, disregarding or distorting the consequences; 7, dehumanization and 8, attribution of blame. These mechanisms are perceived as justifiers for corporate-level safety transgressions [88,89,90,91]. Several other ways to achieve this are found, e.g., to rationalize unethical behaviour [84], to hire people “*who have a greater propensity to morally disengage*” and use them “*to perpetuate organizational corruption*” [87].

Creating a narrow vision of workers on corporate deceit of the public via corporate rhetoric, using incentives (e.g., a bonus) for immoral behaviour, or setting immoral conduct examples in society as a frame of reference [92] all lead to moral disengagement. Encouragement of unethical behaviour in teams is also observed in practice [93].

#### 4.6.4. Workers Risk Exposure

Companies might be presenting employees, third party workers or the general public with unsafe workplaces, health and safety risks or environmental hazards. Allowing the people involved remain ignorant about such risks is often mentioned in literature [46]. This involves, e.g., informing them about the hazardous nature of chemicals [38]. More indirect ways to risk exposure are caused by allowing flaws in ethical infrastructures in companies [84,94] and by acceptance of employee accident underreporting via bureaucratic and technocratic culture [95]. In risk assessments, allowing a lack of risk appetite, and a lack of knowledge, leads to uncontrolled risks on the shop floor [83]. Poor risk awareness, flawed technical- and human intervention-based preventive safety measures, and intangible risk components not dealt with, can affect safety [63,83].

Attempts to find out when doing harm can appear permissible by doing something good at the same time [96] may lead to operations with two goals of which one is immoral, e.g., increasing production by exposing a worker to more danger to increase profit [68]. This unethical type of practice is named the “*doctrine of double effect*” [96].

#### 4.6.5. Fraudulent Practices

There are many ways fraudulent actions can affect safety, and harm people and the environment. Examples are, e.g., bid cutting, bid shopping, bribery, bullying, carelessness, collusive tendering, facilitation payments, corruption, cover pricing, deceptive practices, discrimination, dishonest actuations, dishonest social behaviour, environmental fraud, exert pressure to win at all costs, financial irregularities, artificially inflate profits misrepresentation, negligence, racial discrimination, sexual harassment, under bidding, extortion and withdrawal of tender [52].

### 4.7. Countermeasures

Many of the sources found mention countermeasures. These are presented in a variety of ways and aim at a variety of aspects. Six interconnected groups of measures emerge: 1, ethical management policy; 2, ethical leadership; 3, ethical risk assessment; 4, ethical climate; 5, ethical decision making and 6,ethical behaviour [22,50,97,98]. Table 2 presents an overview of countermeasures as reported from practice, together with references to source information. Each of these measures comprises activities proven to contribute to dedicated risk control, as reported in scientific literature. Together, the measures and activities constitute a set of best practices. It is proposed, as a start, to include this set of six measures and their associated best practices, presented in Table 2, in safety management systems to control the risks associated with misconduct and transgression.

## 5. Discussion

### 5.1. Effect of Misconduct and Transgression on Safety and Health

As Melchers (2001) stated: “The unacceptability need not be in terms of tangible losses only, but can also involve moral or ethical criteria” [5] p. 64.

Although the findings of this study indicate compromised safety performance [4,5] we observe that none of the sources found indicate the existence of any difference between the possible effects on safety, health, the environment, originating from misconduct and transgression, and possible effects originating from other causality, e.g., pollution, occupational disease and exposure to unsafe working conditions.

It seems likely though, that not all of the misconduct and transgression risks and their possible effects would normally be taken into account in a company risk inventory. For safety management this implies that the introduction of new dedicated prevention activities may be important and existing repression activities may not suffice.

### 5.2. Safety versus Security Domain

The proposed way to include measures in safety management systems, as outlined in Table 2, is based on several considerations. One can argue that a safety risk caused by misconduct and transgression both requires law enforcement in some cases (e.g., theft, fraud), and should in its entirety be considered as a security problem rather than a safety problem. The essential difference between these two domains is the presence of a deliberate and conscious intention to inflict physical harm to people and damage property in the security domain, and the lack of such intention in the safety domain [111]. The five groups of misconduct and transgression found in this study, abuse of power, wrong or misleading information, reducing moral awareness, workers risk exposure, and fraudulent practices, imply the opposite since the people involved have other intentions and prefer to go unnoticed.

### 5.3. Safety Management versus General Management Domain

Similar to any other hazard, the safety hazards caused by immoral conduct originate from within the ISO-31000 standard [7] realms of risk control and safety management. Hence, these hazards can be regarded as deviations from, e.g., proper risk assessment, risk analysis, safety culture, an environmental protection system, safe work policy, sound process installation design, routine preventive maintenance, employing skilled personnel and adhering to sustainable business practice. Therefore, it would seem practical to treat the risks associated with immoral and unethical conduct in a similar manner as all other safety risks. This is logical since those other safety risks are also mixed with, e.g., financial, commercial and social aspects, dealt with by general management or by other management disciplines. This involves the inclusion of misconduct and transgression safety risks in company risk assessments, starting from the known international case history, taking up the associated scenarios in the company risk inventory and implementing specific preventive and repressive safety measures to control the risks. Hence, this can bring the control of safety risks originating from immoral and unethical conduct within the bounds of safety management systems. Implementation can best be started from the six elements in the generic SMS framework model as developed by Hale [106] and validated by comparison with a range of SMS’s from practice [112]. The approach in this model ensures risk control system performance, learning and generation of performance data by the successive implementation of procedures on 1, business processes; 2, risk inventory; 3, risk barriers; 4, management system; 5, inspection and monitoring; 6, auditing and management review and 7, unwanted event analysis.

### 5.4. Independent Governance

The role of safety management itself, however, might be overruled or compromised in cases where the immoral and unethical conduct originates from the safety management itself, from executive management inside the organisation, from a corporate head-office abroad, from board members, and from influential commercial or financial stakeholders [52,68,76,81]. It is likely that safety managers cannot control the risks of immoral and unethical conduct only by themselves. The other stakeholders involved with a company need to embrace the ethical way of doing business too and take part in the control of these risks. Governance by independent external supervisors and inspection by government regulators needs to safeguard that this is being done.

Assessing whether risk assessment and risk calculation are sincere and ethical, whether risk acceptance is correctly performed via a fair approach (e.g., ALARP) and whether the barriers as installed are actually sufficient to control the risk, is a complex task. Therefore, risk calculations, e.g., applying risk thermostat-based formulas with risk in micromorts, monetized risk and the value of a human life, as they are vulnerable to assumptions, uncertainty, errors, incomplete modelling, neglected non-quantifiable aspects and lack of transparency for internal and external governance, should be performed very carefully since they may raise ethical concerns [110].

## 6. Conclusions and Recommendations

The results of this study show that safety risks, originating from misconduct and transgression, pose a challenge to both safety managers and safety management systems. Safety managers can start to implement six groups of measures which are readily available as the best practices. Company safety management systems then need to be further developed to accommodate a risk control system for safety risks associated with misconduct and transgression. Governance, comprising independent external supervision and government regulator inspection, is needed to safeguard proper functioning of the implemented risk control system.

It is recommended that further research into the safety risks associated with misconduct and transgression is conducted. Guidance for assessment of safety risks, associated with immoral and unethical conduct and corporate transgression, needs to be developed. It is also necessary that an implementation framework is established for embedding risk control measures in existing safety management systems.

## Figures and Tables

**Table 1:** Ethical principles and emerging corporate conduct principles relevant to health and safety.

Ethical Principles in Society	International Institutions Proposed Ethical Principles	Emerging Corporate Ethical Conduct Standard Principles
**Responsibility**	**No corruption**	**1. Fiduciary principle**
Take responsibility for corrective action on unsafety, misconduct and transgression.	Avoid illegal activities and corruption. Respect laws, rules, conventions and codes of conduct.	Act to prohibit unethical activities.
**Reparation**		**2. Property principle**
Undo damage done to others.		Respect intellectual and physical property, no theft, no illegal waste.
**Integrity/Reciprocity, Golden rule**		**3. Reliability principle**
Treat others as you like to be treated, honesty, keep promises (fidelity), loyalty, commitment.		Be trustworthy, keep promises, abide by contracts.
**The common good**		**X**
Common good is preferable over the good for an individual or organization.		
**Cautionary principle**	**Criteria for risk acceptance**	**X**
Use caution or avoid the action when extreme consequences are involved.	Use methods and criteria for risk analysis and acceptance decision making.	
**Precautionary principle**	**Respect the environment**	**X (see 7.)**
If the effect of an action can be dangerous and uncertain, take preventive measures, or do not do the action.	Precautionary approach of environment, responsibility for environment, use environmentally friendly technology.	
**Transparency principle**	**Transparency**	**4. Transparency principle**
Provide good information, e.g., about risks.	Transparent risk communication.	Respect for truth, accurate information, display openness.
**Autonomy**		**X**
Protect people’s autonomy and decisions, (risk-) informed consent.		
**Value for human life**		**5. Dignity principle**
Protecting human lives is more important than goods. An action should be based on caring for the people and their relationships.		Respect for people, health, safety, rights and privacy.
**Do no harm/Non-maleficence/Value for human life/Non-injury**	**Protection of human rights**	**X (see 5. and 6.)**
Do no harm. Protect people’s health and safety, nature and the environment.	Protection of human rights, non-complicity in abuse, free association, no forced labour, no child labour, no discrimination.	
**Justice (as fairness) principle/Rights principle**		**X (see 5. and 6.)**
Equal people should be treated equally, unequal people should be treated unequally in proportion to their inequality. An action may be performed only when having the moral right to do so, while not infringing the rights of others.		
**Fairness principle/Distributive justice**		**6. Fairness principle**
The benefit and the burden as the effects of an action should be fairly distributed.		Fair distribution of rewards and burdens, equal pay for equal work, no discrimination.
**Equity principle**	**Equity**	**X**
An activity must be beneficial to all.	An activity must be beneficial to all.	
**Beneficence**	**Respect for societal development**	**7. Citizenship principle**
Help to improve the situation of others, set societal goals or assume societal responsibility or duty. Minimizing harm, seek benefits, balancing them against risks and costs.	Contribute to economic and social development, support responsible globalization.	Respect for and contribution to society and environment. Corporate social responsibility (CSR).
**Excellence principle/Self-improvement**	**Excellence principle**	**X**
Act on possibilities to improve.	Perform beyond the requirements by law, improve and build trust.	Perform beyond the requirements by law [61].
**Virtue principle**		**X (see 7.)**
An action should be based on virtues (e.g., respect for human life, privacy, dignity, freedom, fairness, truth, reciprocity, safety, security, common good, altruism, generosity, tolerance, honesty, wisdom) rather than on vices (e.g., violence, greed, slander, lust, exploitation, intolerance, selfishness).		
**Gratitude**	**Respect stakeholders**	**8. Responsiveness principle**
When appropriate show gratitude.	Stakeholders are to be respected beyond shareholders.	Respond to stakeholder concerns Respect stakeholders [61].

**Table 2:** Misconduct and transgression risk control measures, best practices.

Risk Control Measures Description	References
**1-ETHICAL MANAGEMENT POLICY**	
Include ethics in the company safety policy.	[98]
Ensure full commitment to safety and to ethical principles throughout the organization.	[98]
Embrace philanthropy; adopt the pyramid of corporate social responsibility (CSR).	[22,98,99]
Embrace transparency, e.g., choose for honesty and trust about risks.	[22,46,59,76,100]
Do no harm; protect health and safety, show performance w.r.t. people, planet and profit.	[100,101]
Do things right the first time and do the right things.	[100]
Organize a support system to facilitate ethics management and use ethical policies, codes of conduct, rituals, stories, language, rewards, climate and culture. Evaluate these regularly.	[19,84,98]
Include ethics in human resource management and the selection process.	[22,84]
**2-ETHICAL LEADERSHIP**	
Embrace the values of business ethics.	[98]
Place a high value on safety and aim for values-based safety leadership.	[98,102,103,104]
Lead by example, be a role model, inspirator.	[22,104]
Lead in a human way: be accessible, interested in employees, honest. See, hear and inform employees.	[22]
**3-ETHICAL RISK ASSESSMENT**	
Stimulate risk appetite: systematically investigate unknown or intangible risk	[83]
Avoid the use of false information, e.g., fabricated data, incorrect labelling, poor statistics, false descriptions, biased conclusions, flawed administrative methods.	[63,66,82,83]
**4-ETHICAL CLIMATE**	
Educate everyone on how to do everyday business in a moral, ethical and safe way.	[50,75,77,84,105]
Bring personal, professional and organizational ethics in line, using ethical criteria (egoism, benevolence, principle) and levels of analysis (individual, local, cosmopolitan).	[26,97,106,107]
Stimulate a rules/principled and care/wellbeing cantered local ethical climate.	[97,108,109]
Avoid utilitarianism and psychological safety within teams.	[93]
**5-ETHICAL DECISION MAKING**	
Implement an ethical decision-making process	[94]
Provide guidance about values, ethical principles, handling ethical dilemmas, vulnerability for uncertainties and about imposing risk on people, short/long term.	[22,45,59,71,76]
Distinguish between whether the knowledge is insufficient to predict the effect of an action and whether the outcome is immoral or goes against social norms.	[62]
Make use of well proven existing methods, e.g., cost-benefit analysis (CBA), minimum safety criterion, ALARP, individual risk limits.	[62,110]
Apply ethical principles in decision making: -Cautionary principle: if an action has the potential of extreme danger or damage, be cautious or avoid the action; -Precautionary principle: if an action can be dangerous and uncertain take precautionary measures or do not do the action; -Equity principle: An activity must be beneficial to all; -Fairness principle: Benefits and burden must be fairly distributed.	[22,25,59,62]
**6-ETHICAL BEHAVIOUR**	
Distinguish between safety-compliance and safety participation behaviours.	[70]
Stimulate employees to behave in an ethical way. Explain what is unethical. Stimulate both excellent and ethical work performance. Introduce goals, responsibilities, rewards and behavioural norms for individuals and teams.	[22]
Avoid conflicts of interest, unsafe products and dishonest advertisements.	[22]
Respect for stakeholders, e.g., customers, suppliers, shareholders, local community, shared resources, nature and the environment.	[22]
Take responsibility for corrective action on misconduct and transgression, e.g., self-dealing, theft, disrespect for law, stealing intellectual property, improper handling of waste, breaking promises, breach of contract, misleading information, privacy intrusion, disrespect dignity, neglect health and safety, violate human rights, discrimination, improper political activity, bribery, the doctrine of double effect.	[22,96]
Assess, measure and monitor the ethical aspect of behaviour.	[87,106]

## Data Availability

No new data were created or analyzed in this study. Data sharing is not applicable to this article.

## References

[1] Bandura A., Caprara G.V., Zsolnai L. (2000). Corporate Transgressions through Moral Disengagement. J. Hum. Values.

[2] Clarke M. (1990). Business Crime.

[3] Levi M. (1987). Regulating Fraud: White-Collar Crime and the Criminal Process.

[4] Hollnagel E. (2008). Safety Management—Looking Back or Looking Forward. Resil. Eng. Perspect..

[5] Melchers R.E. (2001). On the ALARP Approach to Risk Management. Reliab. Eng. Syst. Saf..

[6] Hale A.R. (2006). Safety Management, What Do We Know, What Do We Believe We Know, and What Do We Overlook?. Tijdschr. Voor Toegep. Arbowet..

[7] BS ISO 31000 Standard, Risk Management—Principles and Guidelines. www.iso.org/obp/ui/#iso:std:iso:31000:ed-2:v1:en.

[8] Smith S.K., Mountain G.A., Hawkins R.J. (2015). A Scoping Review to Identify the Techniques Frequently Used When Analysing Qualitative Visual Data. Int. J. Soc. Res. Methodol..

[9] Byrne J.A. (2016). Improving the Peer Review of Narrative Literature Reviews. Res. Integr. Peer Rev..

[10] Byrne E., Daykin N., Coad J. (2016). Participatory Photography in Qualitative Research: A Methodological Review. Vis. Methodol..

[11] Cronin P., Ryan F., Coughlan M. (2008). Undertaking a Literature Review: A Step-by-Step Approach. Br. J. Nurs..

[12] Wessels R.H.A. (1997). The Importance and Accessibility of Grey Literature. Inf. Prof..

[13] Noah P.D. (2017). A Systematic Approach to the Qualitative Meta-Synthesis. Issues Inf. Syst..

[14] Lindhout P., van der Werff K., Reniers G. (2020). Improving Education and Training of Dutch Major Hazard Control Inspectors: A 15 Years Longitudinal Case Study. Int. J. Environ. Res. Public Health.

[15] ISO 19011:2018 Standard, Guidelines for Auditing Management Systems. www.iso.org/standard/70017.html.

[16] Purdy G. (2010). ISO 31000: 2009-Setting a New Standard for Risk Management. Risk Anal. Int. J..

[17] Provan D.J., Dekker S.W.A., Rae A.J. (2017). Bureaucracy, Influence and Beliefs: A Literature Review of the Factors Shaping the Role of a Safety Professional. Saf. Sci..

[18] Provan D.J., Dekker S.W., Rae A.J. (2018). Benefactor or Burden: Exploring the Professional Identity of Safety Professionals. J. Saf. Res..

[19] Iavicoli S., Valenti A., Gagliardi D., Rantanen J. (2018). Ethics and Occupational Health in the Contemporary World of Work. Int. J. Environ. Res. Public Health.

[20] Yeh M.-J., Liu H.-C. (2018). Comment on Iavicoli et al. Ethics and Occupational Health in the Contemporary World of Work. *Int. J. Environ. Res. Public Health* 2018, *15*, 1713. Int. J. Environ. Res. Public Health.

[21] Palmer C., McShane K., Sandler R. (2014). Environmental Ethics. Annu. Rev. Environ. Resour..

[22] Trevino L.K., Nelson K.A. (2010). Managing Business Ethics: Straight Talk about How to Do It Right.

[23] Ho M.F., Drew D., McGeorge D., Loosemore M. (2004). Implementing Corporate Ethics Management and Its Comparison with the Safety Management System: A Case Study in Hong Kong. Constr. Manag. Econ..

[24] Cazeri G.T., Anholon R., da Silva D., Ordoñez R.E.C., Quelhas O.L.G., Leal Filho W., Santa-Eulalia L.A. (2018). An Assessment of the Integration Between Corporate Social Responsibility Practices and Management Systems in Brazil Aiming at Sustainability in Enterprises. J. Clean. Prod..

[25] Aven T. (2019). The Science of Risk Analysis: Foundation and Practice.

[26] Johnson A. (2012). Safety Ethics-Black and White? Or Shades of Gray?. SH-Saf. Health-Natl. Saf. Counc..

[27] Krause T. The Ethics of Safety, How a Safety Program Can Be the Starting Point for Building an Ethical Organization. www.ehstoday.com/safety/article/21904310/the-ethics-of-safety.

[28] Krause T.R., Hidley J. (2008). Taking the Lead in Patient Safety: How Healthcare Leaders Influence Behavior and Create Culture.

[29] Rahanu H., Georgiadou E., Siakas K., Ross M. (2018). Imperative Ethical Behaviours in Making Systems Development and Deployment Compliant with Health & Safety and Wellbeing. European Conference on Software Process Improvement.

[30] Groot-Kormelink J.G. (2019). Responsible Innovation: Ethics, Safety and Technology.

[31] Resnik D.B. (2007). Responsibility for Health: Personal, Social, and Environmental. J. Med. Ethics.

[32] Wachter J.K., Bird A.J. (2010). Ethical Considerations for the Occupational Safety and Health Professional for Data Collection, Analysis and Interpretation. Applied Quantitative Methods for Occupational Safety and Health.

[33] Longo F., Padovano A., Umbrello S. (2020). Value-Oriented and Ethical Technology Engineering in Industry 5.0: A Human-Centric Perspective for the Design of the Factory of the Future. Appl. Sci..

[34] Beauchamp T.L., Childress J.F. (2009). Principles of Biomedical Ethics.

[35] Whicher D.M., Kass N.E., Audera-Lopez C., Butt M., Jauregui L., Harris K., Knoche J., Saxena A. (2014). Ethical Issues in Patient Safety Research: A Systematic Review of the Literature. J. Patient Saf..

[36] Westerholm P. (2007). Professional Ethics in Occupational Health-Western European Perspectives. Ind. Health.

[37] Resnik D.B. (2012). Environmental Health Ethics.

[38] Schumann P.L. (2001). A Moral Principles Framework for Human Resource Management Ethics. Hum. Resour. Manag. Rev..

[39] Long R. The Ethics of Safety. safetyrisk.net/.

[40] Abma T., Groot B., Widdershoven G. (2019). The Ethics of Public and Service User Involvement in Health Research: The Need for Participatory Reflection on Everyday Ethical Issues. Am. J. Bioeth..

[41] International Collaboration for Participatory Health Research Position Paper 1: What is Participatory Health Research?. http://www.icphr.org/uploads/2/0/3/9/20399575/ichpr_position_paper_1_defintion_-_version_may_2013.pdf.

[42] International Collaboration for Participatory Health Research Position Paper 2: Participatory Health Research: A Guide to Ethical Principals and Practice. www.icphr.org/position-papers.

[43] International Collaboration for Participatory Health Research Position Paper 3: Impact in Participatory Health Research. http://www.icphr.org/uploads/2/0/3/9/20399575/icphr_position_paper_3_impact_-_march_2020__1_.pdf.

[44] Jobin A., Ienca M., Vayena E. (2019). The Global Landscape of AI Ethics Guidelines. Nat. Mach. Intell..

[45] Wright N., Meijboom F.L.B., Sandøe P. (2010). Thoughts on the Ethics of Preventing and Controlling Epizootic Diseases. Vet. J..

[46] Manuel M.E. (2018). Safety and Risk Management Considerations for CSR. Corporate Social Responsibility in the Maritime Industry.

[47] Wenar L., Rawls J. The Stanford Encyclopedia of Philosophy. plato.stanford.edu/entries/rawls/.

[48] Yeoman R., Mueller-Santos M. (2016). Mutuality in Business. Briefing nr 3.

[49] Robertson D.C., Anderson E. (1993). Control System and Task Environment Effects on Ethical Judgment: An Exploratory Study of Industrial Salespeople. Organ. Sci..

[50] Lindhout P., Reniers G. (2017). What About Nudges in the Process Industry? Exploring a New Safety Management Tool. J. Loss Prev. Process. Ind..

[51] Mea W.J., Sims R.R. (2019). Human Dignity-Centered Business Ethics: A Conceptual Framework for Business Leaders. J. Bus. Ethics.

[52] Tamunomiebi M.D., Elechi B.C. (2020). Ethical Managerial Orientations: Emerging Issues. Eur. J. Hum. Resour. Manag. Studies.

[53] United Nations Transforming Our World: The 2030 Agenda for Sustainable Development. https://sustainabledevelopment.un.org/content/documents/21252030AgendaforSustainableDevelopmentweb.pdf.

[54] Montgomery S. Safety Ethics Through One Professional’s Eyes. https://safetymanagementgroup.com/safety-ethics-through-one-professionals-eyes/.

[55] International Labour Organisation (ILO) Declaration on Fundamental Principles and Rights at Work. www.ilo.org/declaration/thedeclaration/textdeclaration/WCMS_716594/lang--en/index.htm.

[56] United Nations General Assembly (1949). Universal Declaration of Human Rights.

[57] EU (2012). Charter of Fundamental Rights.

[58] Chapman A.R. (2017). A “Violations Approach” for Monitoring the International Covenant on Economic, Social and Cultural Rights (ICESCR) 1. Hum. Rights.

[59] Aven T., Renn O. (2018). Improving Government Policy on Risk: Eight Key Principles. Reliab. Eng. Syst. Saf..

[60] Annan K. The Ten Principles of the UN Global Compact. www.unglobalcompact.org/what-is-gc/mission/principles..

[61] CRT Caux Round Table for Moral Capitalism. www.cauxroundtable.org/principles/.

[62] Ersdal G., Aven T. (2008). Risk Informed Decision-Making and Its Ethical Basis. Reliab. Eng. Syst. Saf..

[63] Wachter J.K. (2011). Ethics: The Absurd Yet Preferred Approach to Safety Management. Prof. Saf..

[64] Eckhardt R. (2001). The Moral Duty to Provide Workplace Safety. Prof. Saf..

[65] Wachter J.K., Bird A.J. (2010). Quantitative Aspects of Occupational Safety and Health Systems. Applied Quantitative Methods for Occupational Safety and Health.

[66] Guraya S.Y., London N.J.M., Guraya S.S. (2014). Ethics in Medical Research. J. Microsc. Ultrastruct..

[67] Artvinli F. (2016). The Ethics of Occupational Health and Safety in Turkey: Responsibility and Consent to Risk. Acta Bioeth..

[68] Hughes R.C. (2019). Paying People to Risk Life or Limb. Bus. Ethics Q..

[69] Maslen S. (2019). Safety Management Through Values: A Critical Engagement with the Moral Labor of Disaster Prevention. Saf. Sci..

[70] Griffin M.A., Neal A. (2000). Perceptions of Safety at Work: A Framework for Linking Safety Climate to Safety Performance, Knowledge and Motivation. J. Occup. Health Psychol..

[71] Hansson S.O. (2013). The Ethics of Risk: Ethical Analysis in an Uncertain World.

[72] Vanem E. (2012). Ethics and Fundamental Principles of Risk Acceptance Criteria. Saf. Sci..

[73] Wachter J.K. (2009). Ethics and the Environment, Safety and Health Professional.

[74] Lindhout P., Swuste P., Teunissen G.J., Ale B.J.M. (2012). Safety in Multilingual Work Settings: Reviewing a Neglected Subject in European Union Policymaking. Eur. J. Lang. Policy.

[75] Kim J., Loewenstein J. (2020). Analogical Encoding Fosters Ethical Decision Making Because Improved Knowledge of Ethical Principles Increases Moral Awareness. J. Bus. Ethics.

[76] Van Herten J., Buikstra S., Bovenkerk B., Stassen E. (2020). Ethical Decision-Making in Zoonotic Disease Control. J. Agric. Environ. Ethics.

[77] Carpenter D., James C. (2017). Moral Intensity and Individual State Constructs: Maturing Safety Culture through an Ethical Lens. J. Health Saf. Res. Pract..

[78] Hollnagel E. (2008). Risk + Barriers = Safety?. Saf. Sci..

[79] Akstinaite V., Sadler-Smith E. (2021). Entrepreneurial hubris. World Encyclopedia of Entrepreneurship.

[80] McMurray R., Pullen A. (2020). Morality, Ethics and Responsibility in Organization and Management.

[81] Bandura A., Caprara G.V., Zsolnai L. (2002). Corporate Transgressions. Ethics Econ. Handb. Bus. Ethics.

[82] Croall H. (1989). Who Is the White-Collar Criminal?. Br. J. Criminol..

[83] Lindhout P., Reniers G. (2017). Risk Validation by the Regulator in Seveso Companies: Assessing the Unknown. J. Loss Prev. Process. Ind..

[84] Martin S.R., Kish-Gephart J.J., Detert J.R. (2014). Blind Forces: Ethical Infrastructures and Moral Disengagement in Organizations. Organ. Psychol. Rev..

[85] Probst T.M., Petitta L., Barbaranelli C., Austin C. (2020). Safety-Related Moral Disengagement in Response to Job Insecurity: Counterintuitive Effects of Perceived Organizational and Supervisor Support. J. Bus. Ethics.

[86] Lindhout P., Kingston-Howlett J.C., Reniers G. (2019). Learning from Language Problem Related Accident Information in the Process Industry: A Literature Study. Process. Saf. Environ. Prot..

[87] Moore C. (2008). Moral Disengagement in Processes of Organizational Corruption: The Effect of Moral Disengagement on Unethical Decision Making, Moral Awareness, and Organizational Advancement.

[88] Bandura A., Reich W. (1990). Mechanisms of Moral Disengagement. Origins of Terrorism: Psychologies, Ideologies, States of Mind.

[89] Bandura A. (1990). Selective Activation and Disengagement of Moral Control. J. Soc. Issues.

[90] Bandura A. (1999). Moral Disengagement in the Perpetuation of Inhumanities. Personal. Soc. Psychol. Rev..

[91] Bandura A. (2002). Selective Moral Disengagement in the Exercise of Moral Agency. J. Moral Educ..

[92] Solomon R.C. (2004). Aristotle, Ethics and Business Organizations. Organ. Stud..

[93] Pearsall M.J., Ellis A.P. (2011). Thick as Thieves: The Effects of Ethical Orientation and Psychological Safety on Unethical Team Behavior. J. Appl. Psychol..

[94] Tenbrunsel A.E., Smith-Crowe K. (2008). Ethical Decision Making: Where We’ve Been and Where We’re Going. Acad. Manag. Ann..

[95] Petitta L., Probst T.M., Barbaranelli C. (2017). Safety Culture, Moral Disengagement, and Accident Underreporting. J. Bus. Ethics.

[96] Nelkin D.K., Rickless S.C. (2014). Three Cheers for the Doctrine of Double Effect. Philos. Phenomenol. Res..

[97] Parboteeah K.P., Kapp E.A. (2008). Ethical Climates and Workplace Safety Behaviors: An Empirical Investigation. J. Bus. Ethics.

[98] Zwetsloot G.I., Aaltonen M., Wybo J.L., Saari J., Kines P., De Beeck R.O. (2013). The Case for Research into the Zero Accident Vision. Saf. Sci..

[99] Carroll A.B. (1991). The Pyramid of Corporate Social Responsibility: Toward the Moral Management of Organizational Stakeholders. Bus. Horiz..

[100] Zwetsloot G.I.J.M. (2003). From Management Systems to Corporate Social Responsibility. J. Bus. Ethics.

[101] Smith J., Dubbink W. (2011). Understanding the Role of Moral Principles in Business Ethics: A Kantian Perspective. Bus. Ethics Q..

[102] Kapp E.A. (2012). The Influence of Supervisor Leadership Practices and Perceived Group Safety Climate on Employee Safety Performance. Saf. Sci..

[103] Thomas T., Schermerhorn Jr J.R., Dienhart J.W. (2004). Strategic Leadership of Ethical Behavior in Business. Acad. Manag. Perspect..

[104] Angulo G. (2020). How Ethical Leadership Influences Moral Disengagement and Encourages Employees to Engage in Ethical Practices. Ph.D. Thesis.

[105] Swidler A. (1986). Culture in Action: Symbols and Strategies. Am. Sociol. Rev..

[106] Cullen J., Victor B., Stephens C. (2001). An Ethical Weather Report: Assessing the Organization’s Ethical Climate. Organ. Dyn..

[107] Kapp E.A., Parboteeah K.P. (2008). Ethical Climate & Safety Performance Design Better Programs, Improve Compliance and Foster Participation. Prof. Saf..

[108] Baskin M.E.B., Vardaman J.M., Hancock J.I. (2016). The Role of Ethical Climate and Moral Disengagement in Well-Intended Employee Rule Breaking. J. Behav. Appl. Manag..

[109] Pagliaro S., Lo Presti A., Barattucci M., Giannella V.A., Barreto M. (2018). on the Effects of Ethical Climate (S) on Employees’ Behavior: A Social Identity Approach. Front. Psychol..

[110] Reniers G.L., Van Erp H.N. (2016). Operational Safety Economics: A Practical Approach Focused on the Chemical and Process Industries.

[111] Reniers G., Landucci G., Khakzad N. (2020). What Safety Models and Principles Can Be Adapted and Used in Security Science?. J. Loss Prev. Process. Ind..

[112] Li Y., Guldenmund F.W. (2018). Safety Management Systems: A Broad Overview of the Literature. Saf. Sci..

